# Clinical application of pulmonary vascular resistance in patients with pulmonary arterial hypertension

**DOI:** 10.1186/s13019-021-01696-4

**Published:** 2021-10-20

**Authors:** Jianying Deng

**Affiliations:** Department of Cardiovascular Surgery, Chongqing Kanghua Zhonglian Cardiovascular Hospital, 168# Haier Road, District of Jiangbei, Chongqing, 400015 China

**Keywords:** Pulmonary arterial hypertension, Pulmonary vascular resistance, Pulmonary circulation

## Abstract

Pulmonary arterial hypertension is a type of malignant pulmonary vascular disease, which is mainly caused by the increase of pulmonary vascular resistance due to the pathological changes of the pulmonary arteriole itself, which eventually leads to right heart failure and death. As one of the diagnostic indicators of hemodynamics, pulmonary vascular resistance plays an irreplaceable role in the pathophysiology, diagnosis and treatment of pulmonary arterial hypertension. It provides more references for the evaluation of pulmonary arterial hypertension patients. This article summarizes the clinical application of pulmonary vascular resistance in patients with pulmonary arterial hypertension.

## Introduction

Pulmonary hypertension is a common pulmonary vascular disease, which is caused by various causes of increased pulmonary artery pressure. Pulmonary arterial hypertension (PAH), as the first category in the clinical classification of pulmonary hypertension, refers to increased pressure in the solitary pulmonary artery, while the pressure in the left atrium and pulmonary veins are normal. It is mainly caused by the increase of pulmonary vascular resistance (PVR) caused by the pathology of the pulmonary arterioles itself, and pulmonary hypertension caused by chronic respiratory diseases, chronic thromboembolic diseases and other unknown factors are excluded. Idiopathic pulmonary arterial hypertension is a subtype of PAH, a type of malignant pulmonary vascular disease of unknown cause, which is mainly characterized by progressive increase in PVR [1, 2]. PVR is a static index for evaluating pulmonary circulation hemodynamics. The unequal branch of the pulmonary artery determines the difference in impedance from the main pulmonary artery to the distal vascular bed [3]. PVR was originally based on Poiseuille's law, which describes the pressure loss caused by fluid flowing through a thin tube, which is proportional to the product of volume flow rate, viscosity coefficient and tube length, and inversely proportional to the 4th power of the tube diameter. It is suitable for Newtonian fluids that are incompressible, do not have acceleration, laminar flow and are longer than the pipe diameter [4–6]. But blood is not fluid, and it is not laminar, and the pulmonary blood vessels are also contractible. Therefore, in clinical work, although the PVR formula is commonly used to evaluate pulmonary vascular disease, it is only a static index and has many influencing factors, so clinicians still need to interpret it accurately. This article reviews the application value of PVR in assessing the condition of PAH patients.

## PVR and pathophysiology of PAH

The increase in PVR is closely related to the occurrence and development of PAH. From a pathophysiological point of view, PAH is a type of cardiopulmonary disease that can affect the arterial, venous circulation and right ventricle of the lung. Due to continuous vasoconstriction, excessive pulmonary vascular remodeling, and in situ thrombosis lead to an increase in PVR, which eventually can lead to right heart failure and even death [7, 8]. However, the influencing factors that lead to the acceleration or deterioration of PAH are still unclear, and may be related to genetics and the combined effects of multiple influencing factors. The influencing factors mainly include the process of vasoconstriction and promotion of remodeling, inflammation, vasoactive molecules, anti-apoptosis, and autoimmune mediators, as well as the interaction of cells and cells, cells and substrates, etc. [9].

From a pathological point of view, PAH is a progressive pulmonary vasculature disease, which is mainly characterized by the proliferation of various types of vascular wall cells (including endothelial cells, smooth muscle cells and fibroblasts) and then develops into a wide range of pulmonary vascular remodeling, which ultimately leads to an increase in PVR [10–13]. The increase in PVR is mainly caused by pulmonary artery contraction and complex arterial damage. Among them, pulmonary artery contraction damage includes media hypertrophy and intima and adventitia thickening, which may be related to the imbalance of different types of cell proliferation and apoptosis in the blood vessel wall. However, complex arterial damage is manifested as plexiform lesions, dilated lesions and arteritis. These changes are considered to be signs of the rapid progress of PAH [14, 15].

## PVR on diagnostic value of PAH

The diagnosis and classification of PAH are complicated and mainly rely on hemodynamic indicators. Right-sided heart catheterization (RHC) is the "gold standard" for the diagnosis of PAH, and it is also an important means of differential diagnosis, evaluation of the condition and treatment effect. As one of hemodynamic diagnostic indicators, PVR has important value. PVR comes from calculations, PVR = (mPAP-PAWP)/CO, where mPAP is the mean pulmonary artery pressure, PAWP is the pulmonary arteriole wedge pressure, and CO is the cardiac output.

### PVR on hemodynamic classification of PH

Pulmonary hypertension is defined as mPAP ≥ 25 mmHg (1 mmHg = 0.133 kPa) measured by the RHC at sea level and resting (Table 1). Including precapillary, postcapillary and mixed pulmonary hypertension (pulmonary artery and pulmonary vein pressure can be increased) [1]. Precapillary pulmonary hypertension includes PAH pulmonary hypertension caused by respiratory diseases and/or hypoxia; pulmonary hypertension caused by chronic pulmonary artery obstruction and pulmonary hypertension caused by unknown causes. The hemodynamics of precapillary pulmonary hypertension were defined as mPAP ≥ 25 mmHg, PAWP ≤ 15 mmHg and PVR > 3 wood units measured by RHC. Postcapillary pulmonary hypertension refers to pulmonary hypertension caused by left heart disease and pulmonary hypertension caused by unknown factors. The hemodynamics of postcapillary pulmonary hypertension is defined as the RHC measuring mPAP ≥ 25 mmHg, PAWP > 15 mmHg, in which simple postcapillary pulmonary hypertension PVR ≤ 3 wood units, mixed postcapillary pulmonary hypertension PVR > 3 wood units. Simonneau and Montani et al. [16] proposed a new definition of pulmonary hypertension hemodynamics. It is recommended to change mPAP ≥ 25 mmHg to ≥ 20 mmHg, and other indicators remain unchanged. Among them, mPAP is defined as a mild increase in 21–24 mmHg. PVR > 3 wood units, although relatively conservative, can more accurately diagnose precapillary and postcapillary pulmonary hypertension, and can distinguish increased pulmonary artery pressure caused by pulmonary vascular disease or increased PAWP. Thenappan et al. [17] found that compared with patients with PAH, patients with pulmonary hypertension caused by left heart disease had lower mPAP and PVR, higher CO, and enlarged left atrium. Studies on pulmonary hypertension caused by respiratory diseases showed that when the pulmonary artery pressure increased significantly, the PVR also increased significantly [18, 19]. Even if the pulmonary artery pressure increased only slightly, the increase in PVR would be greater than 3 wood units. Similar to the results of a prospective study on chronic thromboembolic pulmonary hypertension [20]. The increase in PVR is mainly due to alveolar hypoxia or thrombotic obstruction leading to vasoconstriction or pulmonary vascular remodeling. However, some studies have shown that PVR has no significant difference in responsiveness to hypoxia or hyperoxia in a short period of time [21, 22], and the significant increase in PVR may be related to a decrease in CO or other diseases. In short, PVR is an important indicator for diagnosing PAH, and we need to accurately interpret the data measured by the RHC.

**Table 1:** PVR on hemodynamic classification of PH

Classification	mPAP (mmHg)	PAWP (mmHg)	PVR (wood)
PH	≥ 25		
Precapillary PH (including PAH)	≥ 25	≤ 15	> 3
Postcapillary PH	≥ 25	> 15	
Simple	≥ 25	> 15	≤ 3
Mixed	≥ 25	> 15	> 3

*PH* pulmonary hypertension, *PAH* pulmonary artery hypertension, *mPAP* mean pulmonary artery pressure, *PAWP* pulmonary arteriole wedge pressure, *PVR* pulmonary vascular resistance. 1 mmHg = 0.l33 kPa

### PVR on preoperative assessment of PAH related to congenital heart disease

PAH associated with congenital heart disease is an important subtype of PAH, a type of precapillary pulmonary hypertension, and the most common type of PAH patients in China. Clinical classification of PAH associated with congenital heart disease as follows: (1) Eisenmenger syndrome: including all large defects inside and outside the heart, starting from systemic to pulmonary shunt, and then reversal (pulmonary circulation to systemic shunt) or bidirectional shunt as the PVR is severely elevated, and the patient has cyanosis, secondary to polycythemia and multiple organ involvement. (2) Systemic to pulmonary shunt congenital heart disease: It can be divided into two types: correctable and uncorrectable type. The correctable type refers to a moderate to large defect, PVR is slightly to moderately elevated, and shunts from systemic circulation to pulmonary circulation are still present, and cyanosis is not the main feature. Uncorrectable type means that there is currently no indication for interventional closure and surgical repair. (3) PAH with small defects: small defects combined with a significant increase in PVR (small defects refer to adult ventricular septal defect < 1 cm and atrial septal defect < 2 cm measured by echocardiography), and there is an increase in PVR that cannot be explained by the defect. The clinical features are similar to idiopathic pulmonary hypertension, and such defects cannot be closed. (4) PAH after correction of congenital heart disease: PAH persists after interventional closure or surgical repair of congenital heart disease, or PAH reappears after several months or years, and the condition of such patients often worsens [1, 2].

According to the European Adult Congenital Heart Disease Survey, the overall prevalence of PAH in adult patients with congenital heart disease is 4–28%, and Eisenmenger syndrome is 1–6%. Among all patients with open defects, 34% of patients with atrial septal defect and 28% of patients with ventricular septal defect have PAH, and the corresponding proportions of patients with closed defects are 12% and 13% respectively [23, 24]. PAH associated with congenital heart disease is different from other types of PAH, and has early reversible and late irreversible characteristics; timely closing of the shunt channel can make the pulmonary vessel morphology and hemodynamics normal [25–27]. In addition to considering the nature and size of the defect, the timing of closing the defect and preoperative hemodynamics are also critical. Studies have shown that PVR can become normal when the defect is closed by surgery within 1 year old; PVR may decline after the operation when it is over 2 years old, but it may not return to normal levels [27]. In clinical work, for patients with shunts, the Fick method is often used to measure the pulmonary circulation (Qp)/systemic circulation (Qs) blood flow, and calculate the PVR after quantifying the shunt to assess the feasibility of surgery. Because when there is a large systemic circulation to the pulmonary circulatory shunt, the increase in pulmonary blood flow causes a significant increase in pulmonary artery pressure, and the increase in PVR may not be significant. A retrospective study found that when the baseline value is PVR ≥ 5 wood units, pulmonary vascular resistance index (PVRi) ≥ 6 wood units·m^2^, and the PVR/system-wide vascular resistance ratio ≥ 0.33, after closing the defect of patients, PAH is more likely to occur in the late follow-up stage [28], but this conclusion needs further verification.

At present, the criteria for judging the feasibility of surgery for congenital heart disease combined with PAH are not uniform. According to the available data, the 2015 European Society of Cardiology (ESC)/European Respiratory Society (ERS) Guidelines for Diagnosis and Treatment of Pulmonary Hypertension proposes the criteria for shut-off systemic-pulmonary shunt based on PVR (Table 2): PVR < 2.3 wood units, PVRi < 4 wood units·m^2^, it is recommended to close the defect; when PVR > 4.6 wood units, PVRi > 8 wood units·m^2^, it is not recommended to close the defect; PVR is 2.3 ~ 4.6 wood units, PVRi is 4–8 wood units·m^2^ is the “gray zone”, which requires individualized assessment in a tertiary hospital [1]. For patients in the "gray zone", it is suggested that the defect can be closed when the PVR is reduced to an acceptable level by treating PAH [29, 30], but considering the impact on the right ventricle, we believe that the existing data does not yet support the concept of "treatment to closing defects".

**Table 2:** PVR on surgery criteria of PAH related to congenital heart disease

Recommend defect closing	PVR (wood)	PVRi (wood units · m^2^)
Y	< 2.3	< 4
N	> 4.6	> 8
Y/N*	2.3–4.6	4–8

*PAH* pulmonary artery hypertension, *PVR* pulmonary vascular resistance, *PVRi* pulmonary vascular resistance index, *Y* indicated closing defect, *N* indicated not closing defect

*Indicated requires individualized assessment

### PVR measured by echocardiography on PAH

The diagnosis and treatment process of PAH is becoming standardized, and echocardiography as its non-invasive diagnostic tool can initially screen patients with PAH. It is believed that the use of echocardiography to assess pulmonary artery pressure is reliable. In the absence of pulmonary outflow tract obstruction, the tricuspid regurgitation peak velocity (TRV) is linearly positively correlated with mPAP and the pulmonary artery systolic pressure (sPAP) measured by RHC; while the acceleration time of the right ventricular outflow tract is linearly negatively correlated with sPAP and mPAP [31, 32]. In clinical practice, TRV is mostly used to assess right ventricular systolic pressure (RVSP). On the premise of excluding right ventricular outflow tract obstruction, sPAP is equivalent to RVSP plus right atrial pressure (RAP), RVSP = 4 × (TRV)^2^, sPAP = 4 × (TRV)^2^ + RAP [33, 34]. Echocardiography can also be used to assess PVR, and PVR = TRV (m/s)/TVIRVOT (cm), and the time-velocity integral of the right ventricular outflow tract short for TVIRVOT [35–38]. At present, these assessment methods are based on the recognition that PVR is directly related to pressure changes and negatively related to pulmonary blood flow. Although different calculation methods have proved that there is a certain correlation between the echocardiographic assessment of PVR and the PVR calculated by RHC, further research is needed to confirm. PVR not only helps to distinguish the increase in mPAP caused by increased pulmonary blood flow (such as hyperthyroidism, anemia, obesity, etc.), but also helps to distinguish PAH patients with severe or worsening clinical symptoms but not obvious changes in mPAP [31]. Studies [39, 40] have found that echocardiography may underestimate or overestimate sPAP, and mPAP cannot be directly measured, nor can it indicate whether sPAP is related to pulmonary vascular disease. Therefore, it is not possible to formulate a treatment plan based on clinical manifestations and echocardiographic examination results alone, and RHC examination must be performed.

## PVR on PAH risk stratification, prognosis assessment and early diagnosis

### PVR on risk stratification of PAH

The diagnosis of PAH relies on hemodynamic data, but the evaluation of the condition and prognosis requires reference to multiple clinical indicators. The main indicators in the currently recommended risk stratification table include the World Health Organization (WHO) cardiac function classification, 6-min walking distance (6MWD), N-terminal pro-B-type natriuretic peptide (NT-proBNP), right atrial pressure (RAP), heart index (CI), and mixed venous oxygen saturation (SVO_2_). Studies have shown that PVR and the above indicators are correlated at baseline and 6 months after treatment [41, 42]. Studies on the treatment of connective tissue-related PAH with riociguat showed that the improvement of PVR is consistent with the improvement of 6MWD, WHO cardiac function classification and CI [43, 44]. Simpson et al. [45] related research results on biomarkers showed that NT-proBNP is correlated with RAP, mPAP, cardiac output (CO), and PVR. Although PVR is not included in the risk stratification table, it has a certain correlation with PAH risk stratification related indicators; at the same time, the risk stratification table is only applicable to adult PAH; however, studies have shown that PVR is in adolescents and children similar to adults, the PVR of adolescents is usually less than 2 wood units [46, 47].

### PVR on prognosis of PAH patients

We usually guide and evaluate the treatment and prognosis of PAH patients based on clinical manifestations, echocardiography, and risk stratification related indicators. Most studies have confirmed that CO or CI and RAP at baseline can be used as prognostic factors for PAH [48, 49], while the value of routinely measured mPAP and PVR for prognosis has not yet reached a consensus. Studies on the 1-year survival rate of PAH patients show that mPAP is not an independent predictor of PAH prognosis, while elevated RAP and PVR > 32 wood units are its independent predictors [50]. However, a study [51] on the prognostic value of hemodynamic indicators in PAH patients showed that all-cause death and lung transplantation were used as the research endpoints, and age, gender (male), etiology (idiopathic pulmonary hypertension), New York Heart Association cardiac function classification and 6MWD are independent predictors, and have nothing to do with baseline hemodynamic indicators.

### PVR on early diagnosis of PAH

PVR can be used for early diagnosis of PAH. PVR > 3 wood units, although it is of great significance for the diagnosis of precapillary pulmonary hypertension. However, early CO in many patients tends to be normal and rarely shows PVR > 3 wood units, which cannot meet the diagnosis of precapillary pulmonary hypertension and is not conducive to the early diagnosis of PAH. A study on the hemodynamic characteristics and survival rate of patients with systemic sclerosis (SSc), in addition to showing that PVR and 6MWD can be used as independent predictors of the prognosis of PAH patients, it is also recommended to adjust the PVR cutoff value ≥ 2 wood unit [52]. Studies have found that PVR ≥ 2 wood units are associated with pulmonary vascular disease and the survival rate of patients is significantly reduced, while PVR ≥ 2 wood units are more suitable for early diagnosis of SSc-PAH in high-risk populations. Previous studies [53, 54] have found that dynamic PVR can be used as an independent predictor of SSc-PAH, but its predictive value needs to consider disease classification or progression. PVR is calculated based on the data measured by RHC in the resting state, but dynamic PVR is expressed by the slope of the multipoint mPAP-flow diagram, which can better display the PVR situation, but the clinical significance is still unclear [55, 56].

## Influencing factors of PVR

PAH patients mainly die of right heart failure, and right heart failure is different from right ventricular dysfunction (RVD). Right heart failure is a clinical syndrome with symptoms and signs of heart failure caused by RVD. The most common cause of chronic right heart failure is pulmonary hypertension caused by left heart failure, leading to increased right ventricular afterload [57]. RVD is manifested as an abnormal structure or function of the right ventricle, and right ventricular systolic dysfunction (RVSD) is an important predictor of the prognosis of heart failure. Studies have shown that in addition to heart failure with reduced ejection fraction, RVSD is also more common in heart failure with preserved ejection fraction and has a poor prognosis [58]. Studies have confirmed that in patients with heart failure with preserved ejection fraction, the baseline level of PVR in RVSD patients is significantly increased, the right ventricular ejection fraction is related to PVR but not mPAP, and both RVSD and PVR have an impact on the prognosis [59, 60]. Badagliacca et al. [61] found that PVR reduction is the only determinant of right ventricular reversal remodeling, and proposed that the reason why PVR improvement cannot be an independent predictor of prognosis may be because its reduction is only a driving factor for the improvement of right ventricular structure and function.

Roca et al. [62] in the study of the effect of reduced PVR on the left and right ventricular changes in PAH patients found that baseline PVR was significantly related to the degree of longitudinal muscle fiber damage in the free wall of the right ventricle, but not with the left ventricular changes. The decrease in PVR at 24 weeks of treatment was related to the improvement of the left and right ventricles. Right heart failure is the main cause of death in PAH patients, and the increase in right ventricular afterload is mainly determined by the pressure-flow relationship. However, PVR cannot fully explain the right ventricular afterload, because it ignores the impact of pulsatile load [63]. As early as 1682, William Harvey discovered the interaction between the right ventricle and the pulmonary circulation, but then the concept of "right ventricle-pulmonary artery" coupling was not taken seriously. In recent years, with the improvement of PAH awareness, people have begun to pay attention to the overall concept of right ventricle-pulmonary artery. It has also been proposed that pulmonary vascular impedance can more accurately describe the pressure-flow relationship, because the combined effect of PVR and total pulmonary artery compliance (PCa) is considered, but there are still difficulties in its measurement and interpretation [3]. Ideally, we think that the two are separable indicators, that is, when the PVR increases or decreases in the pulmonary circulation, the total PCa decreases or increases accordingly. However, some studies have found that PVR is closely related to age. Although PVR is low in elderly patients, PCa does not increase correspondingly, and it is believed that PCa has a greater impact on its prognostic value [64, 65]. A study [66] found that in patients with precapillary pulmonary hypertension, PVR may be overestimated according to the formula calculation, because the average lung blood flow is represented by the transpulmonary pressure difference, that is, mPAP minus the pressure at the average downstream zero flow; generally use PAWP instead, but the actual PAWP is less than the average downstream pressure at zero flow.

In addition to calculation errors, there are many other factors that affect PVR. PVR is negatively correlated with total PCa and also negatively correlated with pulmonary vessel volume [67]. According to the definition of PVR, it is related to blood viscosity, that is, hematocrit can also affect PVR [68]. At the same time, with the understanding of exercise-related pulmonary hypertension, it is confirmed that PVR is different between rest and exercise [69]. Some researchers also believe that preterm birth may increase PVR. Studies have found that young people with a history of preterm birth may have pulmonary vascular disease, which is manifested by increased mPAP, increased PVR during rest and exercise (pulmonary capillary bed stiffness), decreased CO during exercise and decreased right ventricular systolic function [70]. Some researchers have found that air pollution may also increase PVR by reducing CO [71]. In short, a more comprehensive understanding of the influencing factors of PVR helps to accurately interpret it.

## Conclusions and outlook

In summary, PAH is a rare but serious pulmonary vascular disease. As one of the diagnostic indicators of PAH hemodynamics, PVR plays an irreplaceable role in the diagnosis and clinical classification of precapillary pulmonary hypertension. At the same time, PVR and PAH risk stratification indicators have a certain correlation, and also have important value in the prognosis and early diagnosis of some PAH. However, it is a static index and does not include pulsatile load. It is not the best index for evaluation of right ventricular afterload. Pulmonary circulation is different from systemic circulation because it is a vascular system with low resistance and high compliance. Considering the heterogeneity of PAH, we should not only have a overall concept of "right ventricle-pulmonary artery", but also explore the value of "pulmonary vascular impedance". I believe that with the development of medical technology, we will have a better understanding of PAH.

## Data Availability

All data can be contacted with the authors.

## Abbreviations

PAH: Pulmonary arterial hypertension
PH: Pulmonary hypertension
PVR: Pulmonary vascular resistance
RHC: Right-sided heart catheterization
mPAP: Mean pulmonary artery pressure
PAWP: Pulmonary arteriole wedge pressure
CO: Cardiac output
Qp: Pulmonary circulation
Qs: Systemic circulation
PVRi: Pulmonary vascular resistance index
ESC: European Society of Cardiology
ERS: European Respiratory Society
TRV: Tricuspid regurgitation peak velocity
sPAP: Pulmonary artery systolic pressure
RVSP: Right ventricular systolic pressure
RAP: Right atrial pressure
TVIRVOT: Time-velocity integral of the right ventricular outflow tract
WHO: World Health Organization
6MWD: 6-Min walking distance
NT-proBNP: N-terminal pro-B-type natriuretic peptide
CI: Heart index
SVO_2_: Mixed venous oxygen saturation
SSc: Systemic sclerosis
RVD: Right ventricular dysfunction
RVSD: Right ventricular systolic dysfunction
PCa: Pulmonary artery compliance
